# Analysis of risk factors for the development of obesity in preschool children: a logistic model analysis

**DOI:** 10.3389/fped.2025.1497962

**Published:** 2025-01-28

**Authors:** Huasheng Jin, Huihui Zhou, Kai Chen

**Affiliations:** Department of Pediatrics, Ningbo Yinzhou No.2 Hospital, Yinzhou, Ningbo, Zhejiang, China

**Keywords:** obesity, preschool children, risk factors, interventions, pediatric health

## Abstract

**Introduction:**

Obesity, characterized by abnormal fat accumulation, has become a significant global health concern. The prevalence of obesity in preschool children is rising across regions, highlighting the need for a thorough analysis of risk factors and effective interventions to address this growing epidemic. The aim of this study was to identify and analyze the main risk factors for the development of obesity in preschool children and explore effective interventions.

**Methods:**

We collected clinical data from 910 preschool children who underwent a physical examination at our hospital from January 2023 to December 2023. Based on specific inclusion and exclusion criteria, we categorized the children into two groups: the obesity group (149 cases) and the non-obesity group (761 cases). Using univariate analysis and logistic regression modeling, we identified risk factors associated with obesity development.

**Results:**

Out of the 910 children, 149 were diagnosed as obese, representing 16.37% of the total. High birth weight, high parental body mass index (BMI), picky eating habits, preference for meat, frequent consumption of junk food, and binge eating were found to be significant risk factors for obesity in preschool children. Meanwhile, higher parental education levels and longer daily outdoor activity times were considered protective factors against obesity.

**Discussion:**

Effective interventions targeting the above risk factors are necessary to minimize the incidence of obesity and promote the healthy development of children.

## Introduction

Obesity is globally recognized as a complex chronic disease characterized by the abnormal accumulation of body fat, which poses significant harm to health (1, 2). With the rapid socioeconomic development, the incidence of obesity in children has been increasing, and childhood obesity has gradually become an important public health issue, attracting widespread attention (3). The widely used international measure of obesity is the body mass index (BMI), calculated by dividing weight (in kg) by the square of height (in m) (4). The World Health Organization (WHO) provides percentile charts for BMI, categorized by age and gender, to assess children's weight status (5).

The prevalence of childhood obesity exhibits diverse growth patterns across different regions. In the United States, the prevalence of obesity among 2- to 19-years-old increased from 17.7% in 2011–2012 to 21.5% in 2017–2020 (6). A study conducted across multiple European countries revealed that the overall obesity prevalence among children aged 6 to 14 years was 17.55% from the early 2000s to 2020. During the first decade, the obesity prevalence ratio for girls was 4.76, while for boys, it was 5.26. In the second decade, these ratios increased to 5.96 for girls and 6.94 for boys (7). Since the 1980s, the prevalence of obesity in Chinese children has also rapidly increased. The survey findings from students aged 7–18 across 30 provinces revealed that the prevalence of overweight and obesity stood at 7.7% and 3.6% respectively in 2000. By 2010, these rates had increased to 12.7% for overweight and 7.3% for obesity, effectively doubling over the span of a decade (8).

Recent studies have identified several risk factors associated with childhood obesity, which can be categorized into genetic, environmental, and behavioral factors. While genetic factors play a role in determining an individual's propensity for fat accumulation and energy metabolism, environmental and lifestyle factors, especially unhealthy diets and lack of physical activity, often act as key contributors to the development of obesity (9). The rise of fast-food culture, the increased consumption of high-sugar and high-fat foods, and the growing use of electronic devices have all been linked to the rising rates of obesity (10). Furthermore, family environment and parents' health behaviors significantly influence children's obesity risk (11).

Given the extensive impact of obesity on children's physical and mental health, as well as its increasingly severe epidemic situation in different regions, analyzing the risk factors for obesity is particularly important. This study aims to provide a more focused understanding of childhood obesity in China through a systematic analysis of risk factors among preschool children, with the acknowledgment that the findings may have limited generalizability beyond this specific population. Additionally, it aims to explore effective prevention and intervention measures to promote the health and well-being of children.

## Objects and methods

### Objects

A total of 910 preschool children who underwent physical examination in our hospital from January 2023 to December 2023 were selected for the study. Inclusion criteria: (1) All preschool children aged 3–7 years; (2) No intellectual development abnormalities, able to communicate simple information; (3) Parents or legal guardians of the children provided informed consent for participation in the questionnaire survey, with children voluntarily agreeing to participate. Exclusion criteria: (1) Children with neurological diseases such as cerebral palsy and epilepsy; (2) Parents with inherited genetic diseases, mental illnesses, hyperthyroidism, kidney diseases, or heart diseases; (3) Refusal to participate in the study or withdrawal during the study. This study was approved by the hospital's Medical Ethics Committee and obtained informed consent from the parents of the children.

## Methods

### Ethical approval

This study was approved by the Ethic Committee of Ningbo Yinzhou No.2 Hospital in Ningbo, Zhejiang Province, China (No. YinErLunShen2024YanPi-068), in accordance with regulatory and ethical guidelines pertaining to retrospective research studies. All procedures followed the ethical standards of the local legislation and institutional requirements and with the Helsinki Declaration. Parents or legal guardians of the children provided informed consent for participation in the questionnaire survey, with children voluntarily agreeing to participate.

### Allocation of participant

The following factors were considered during the group allocation process: ① Age: Participants were grouped according to age brackets to control for any age-related effects on dietary habits and behaviors. ② Gender: the balanced representation of males and females across all groups to control was ensured to avoid any potential gender-related differences in eating behaviors. ③ BMI of Parents: Given the potential influence of parental BMI on children's eating habits, the BMI of both parents when assigning participants to groups were took into account. ④ Socioeconomic Status: family income and parental education level to control for socioeconomic factors that may influence dietary behaviors were considered. ⑤ Pregnancy-related Factors: The mother's weight gain during pregnancy and the delivery method were also factored into the allocation process, as these could influence the child's early nutrition and eating patterns.

### Measurement items

With the help of the parents, medical personnel conducted physical examinations on all children, measuring their height and weight. For height measurement, a Seca 213 portable stadiometer (Seca GmbH & Co. KG, Hamburg, Germany) was used, with an accuracy of 1 mm. After calibrating the instrument, the children were required to remove their shoes and hats, stand in the middle of the height measuring device with their heads held high, eyes looking straight ahead, knees together, heels together, and step back with their feet straight, and then the readings were taken. Two consecutive measurements were taken, and the average of the two results was used as the final measurement result. For weight measurement, a Seca 803 digital scale (Seca GmbH & Co. KG, Hamburg, Germany) was used, with an accuracy of 0.5 kg. After calibrating the instrument, the children were required to empty their bowels before measurement, remove their shoes and stand in the middle of the weighing device, avoiding body movement, and then the readings were taken. Two consecutive measurements were taken, and the average of the two results was used as the final measurement result.

### Assessment criteria

Combined with the growth standards for children formulated by the World Health Organization (12, 13), F = (measured weight—median weight of height and weight)/median weight of height and weight × 100%. For F ≥ 10% but <20%, it was classified as overweight; for F ≥ 20% but <30%, it was classified as mild obesity; for F ≥ 30% but <50%, it was classified as moderate obesity; for F ≥ 50%, it was classified as severe obesity.

### Questionnaire survey

A self-designed questionnaire was used to conduct the survey, covering the following aspects: (1) Basic characteristics of the children: including name, age, gender, whether they are only children, and birth weight; (2) Basic information of the family: including the parents' body mass index (BMI), education level, and family income. In our study, parental education levels were categorized into four distinct groups to reflect the general educational attainment of the parents. The categories were as follows: ① No formal education: Parents who did not receive any formal schooling or completed less than a primary school education; ② Primary/Secondary education: Parents who completed primary school or lower secondary education; ③ High school education: Parents who completed high school or its equivalent; ④ Tertiary education: Parents who completed post-secondary education, including vocational training, college, or university degrees; (3) Basic information about the mother during pregnancy: including weight gain during pregnancy and delivery method; (4) Children's dietary habits: mainly including the duration of breastfeeding, preference for meat, vegetables, beans, and milk, frequency of junk food intake (including fried foods, carbonated drinks, and puffed food), the presence of picky eating, and binge eating habits. The questionnaire contained 30 questions, and the following questions were specifically used to assess dietary habits: ① “How often do you consume fried foods?”; ② “How often do you consume carbonated drinks?”; ③ “How often do you consume puffed foods?”; ④ “Do you have any picky eating habits?”; ⑤ “Do you engage in binge eating?” (In this study, “junk food” refers to foods that are high in sugar, fat, and low in nutritional value. Specifically, this includes fried foods, carbonated drinks, puffed foods, chips, candies, and other highly processed foods); (5) Physical activity and screen time: including daily screen time, daily sleep duration, and daily duration of outdoor physical activity. Physical activity was defined as time spent engaging in outdoor play, sports, or other activities such as running, cycling, or playing outdoor games. Screen time included time spent on activities such as watching TV, playing video games, and using electronic devices.

### Survey methods

The above questionnaire was used for the survey. Under the guidance of trained personnel, all questionnaires were filled out by the parents of the children. The parents were required to fill out the questionnaires strictly according to the actual situation. After filling out the questionnaires, the staff checked for any omissions or errors, and then collected them. A total of 910 questionnaires were distributed in this study, and 910 valid questionnaires were collected, with a recovery rate of 100%.

### Observation indicators

The differences in the data from the two groups of questionnaire surveys were observed, and single-factor analysis was conducted. The items with differences in the single-factor analysis were brought into the Logistic regression equation to determine the main risk factors for the development of obesity.

### Statistical methods

Data analysis was conducted using SPSS 25.0 statistical software. Count data was represented as [n(%)] and the chi-square test was used with the basic formula if the sample size (n) ≥ 40 and the expected frequency (T) ≥ 5. The test statistic was *χ*^2^. If the sample size (n) ≥ 40 but the expected frequency (T) was between 1 and 5, the chi-square test with correction formula was used. If the sample size (n) < 40 or the expected frequency (T) < 1, Fisher's exact probability method was used for statistical analysis. For continuous data, we first assessed normality using the Shapiro-Wilk test (for small samples) and the Kolmogorov-Smirnov test (for larger samples). If the data was normally distributed, it was represented as ($\bar{\chi }$±s) and analyzed using the *t*-test. For non-normally distributed data, the median and interquartile range [M (P25, P75)] were reported, and the Mann-Whitney *U*-test (a non-parametric test) was used instead of the *Z*-test, to avoid assumptions of normality. Analysis of variance (ANOVA) was used for comparisons of continuous data among multiple groups. Items with significant differences in single-factor analysis were included in the logistic regression equation, with obesity as the dependent variable and items from the questionnaire as independent variables. The criterion for stepwise variable selection was *P* = 0.05, and factors influencing obesity in preschool children were analyzed, with *P* < 0.05 indicating statistical significance.

## Results

### Basic information

As shown in Table 1, during the survey, a total of 910 valid questionnaires were collected, including 524 boys (57.58%) and 386 girls (42.42%). The mean height of all children was 112.40 cm, with the highest measurement of 134 cm and the lowest measurement of 90 cm; the mean weight was 20.08 kg, with the highest measurement of 53 kg and the lowest measurement of 12 kg. According to the relevant standards formulated by the World Health Organization, a total of 149 cases of obesity were screened, accounting for 16.37% of all children. Among them, there were 80 boys, accounting for 8.79% of all children with obesity, and 69 girls, accounting for 7.58% of all children with obesity. These 149 cases of obesity were classified into three levels based on BMI-for-age percentiles: Overweight: BMI ≥ 85th percentile and < 95th percentile; Obesity: BMI ≥ 95th percentile and < 99th percentile; Severe Obesity: BMI ≥ 99th percentile.

**Table 1:** Obesity Status of preschool children.

Variable		Total (*n* = 910)	Obese group (*n* = 149)	Non-obese group (*n* = 761)
Sex	Male	524 (57.58%)	80 (53.69%)	444 (58.34%)
	Female	386 (42.42%)	69 (46.31%)	317 (41.66%)
Age (years)	Mean ± SD	4.87 ± 1.02	5.1 ± 1.05	4.83 ± 1.01
	Range	3–7	3–7	3–7
Height (cm)	Mean ± SD	112.40 ± 8.56	115.12 ± 9.08	111.87 ± 8.42
	Range	90–134	98–134	90–130
Weight (kg)	Mean ± SD	20.08 ± 6.72	27.14 ± 8.33	18.68 ± 5.32
	Range	12–53	18–53	12–45

### Single-factor analysis of factors affecting the development of obesity in preschool children

As shown in Table 2, the 149 children who developed obesity were included in the obesity group, and the 761 children who did not develop obesity were included in the non-obesity group. There were no statistically significant differences between the two groups in terms of whether they were only children, monthly family income, maternal weight gain during pregnancy, mode of delivery, duration of breastfeeding, daily screen time, and daily sleep time (*P* > 0.05). However, there were statistically significant differences between the two groups in terms of birth weight, paternal BMI, maternal BMI, paternal education level, maternal education level, whether the child was picky eater, preference for meat or vegetables, frequency of junk food intake, and presence of binge eating (*P* < 0.05), suggesting that these factors might be the crucial induction factors in the development of obesity in preschool children.

**Table 2:** Univariate analysis of factors influencing obesity in preschool children.

Variable		Number	Obese group (*n* = 149)	Non-obese group (*n* = 761)	*χ*2	*P*
Birth weight	<4000g	602	54	548	71.199	0.001
	≥4000g	308	95	213		
Whether it is the only child	Yes	696	111	585	0.391	0.532
	No	214	38	176		
Father's body mass index	<24 kg/m^2^	452	52	400	15.55	0.001
	≥24 kg/m^2^	458	97	361		
Father's education level	Junior high school or below	204	47	157	8.306	0.016
	High school	411	59	357		
	Junior college or above	290	43	247		
Mother's body mass index (BMI)	<24 kg/m^2^	504	48	556	55.335	0.001
	≥24 kg/m^2^	406	101	305		
Mother's level of education	Junior high school or below	386	39	347	27.157	0.001
	High school	202	54	148		
	Junior college or above	322	56	266		
Monthly household income	< $460	220	30	190	2.806	0.246
	$460-$770	492	80	412		
	>$770	198	39	159		
Amount of weight gained during pregnancy by the mother	<15kg	578	88	490	1.527	0.217
	≥15kg	332	61	271		
Delivery method	vaginal delivery	581	89	492	1.307	0.253
	cesarean section	329	60	269		
Duration of breastfeeding	less than 6 months	96	16	80	0.014	0.993
	6-12 months	622	102	520		
	more than 12 months	192	31	161		
Whether the child is picky about food	Yes	590	125	465	28.383	0.001
	No	320	24	296		
Prefer meat or vegetables	Meat	354	99	255	56.864	0.001
	Vegetables	556	50	506		
Frequency of intake of junk food	Never or less than once a week	100	10	90	67.466	0.001
	1–3 times per week	496	54	442		
	More than 3 times per week	314	85	229		
Whether there is binge eating	Yes	362	79	283	13.038	0.001
	No	548	70	478		
Daily duration of outdoor physical activity	<30min	235	38	197	7.722	0.021
	30–90min	484	86	398		
	>90min	191	25	166		
Daily screen time	<1h	198	30	168	1.027	0.598
	1–2h	502	80	422		
	>2h	210	39	171		
Daily sleep duration	<8h	448	72	376	0.059	0.808
	≥8h	462	77	385		

### Assignment table for independent variables

As shown in Table 3, birth weight, paternal BMI, maternal BMI, paternal education level, maternal education level, whether the child is picky eater, preference for meat or vegetables, frequency of junk food intake, and presence of binge eating, and daily outdoor exercise time were assigned values according to different criteria.

**Table 3:** Independent variable assignment table.

Independent variable	The assignment method is as follows
Birth weight	Less than 4,000 g is assigned a value of 0, and more than 4,000 g is assigned a value of 1.
Father's body mass index	Less than 24 kg/m^2^ is assigned a value of 0, and greater than or equal to 24 kg/m^2^ is assigned a value of 1.
Mother's body mass index	Less than 24 kg/m^2^ is assigned a value of 0, and greater than or equal to 24 kg/m^2^ is assigned a value of 1.
Father's education level	High school and above is assigned a value of 0, and junior high school and below is assigned a value of 1.
Mother's education level	High school and above is assigned a value of 0, and junior high school and below is assigned a value of 1.
Whether the child has picky eating habits	No is assigned a value of 0, and Yes is assigned a value of 1.
Preference for meat or vegetables	Vegetables are assigned a value of 0, and meat is assigned a value of 1.
Frequency of junk food intake	Never or less than once a week is assigned a value of 0, and once a week or more is assigned a value of 1.
Whether there is binge eating	Nothing is assigned a value of 0, and Something is assigned a value of 1.
Daily outdoor exercise time	More than or equal to 30 min is assigned a value of 0, and less than 30 min is assigned a value of 1.

### Multifactorial analysis of factors affecting the development of obesity in preschool children

As shown in Table 4, the items with differences in the single-factor analysis were brought into the Logistic regression equation, and it was found that high birth weight, high paternal BMI, high maternal BMI, picky eating, preference for meat, high frequency of junk food intake, and binge eating were all risk factors for the development of obesity in preschool children, with all results like odds ratios (ORs) shown in Table 4.

**Table 4:** Multifactorial analysis of factors influencing the development of obesity in preschool children.

Factors	*β* value	SE	*P*	Wald value	*OR* value	*OR* value 95%*CI*
High birth weight	0.745	0.870	0.001	12.262	2.674	2.014–2.885
High father's body mass index	0.698	0.744	0.001	13.248	1.735	1.695–1.871
High mother's body mass index	0.674	0.728	0.001	9.410	1.606	1.186–2.288
High father's education level	0.825	0.914	0.001	15.732	0.404	0.348–0.530
High mother's education level	1.168	0.404	0.035	8.366	0.385	0.325–0.478
Child picky eater	1.067	0.424	0.020	6.350	1.078	1.020–1.128
Fonder of meat	1.175	0.412	0.001	8.119	2.154	1.982–2.260
High frequency of intake of junk food	1.500	0.415	0.001	13.072	2.098	2.004–2.186
Binge eating	2.918	0.459	0.001	40.337	1.865	1.802–1.974
Long daily outdoor exercise time	0.825	0.914	0.028	15.732	0.465	0.308–0.680

β, regression coefficient; SE, standard error; OR, odds ratio; CI, confidence interval.

## Discussion

With the progress of the domestic economy and the improvement of people's living standards, the rate of obesity in children has also increased, and the harm of obesity to children's physical and mental health can continue into adulthood (14). After the occurrence of obesity, the proportion of fat in the body is relatively high, which often leads to complications such as coronary heart disease, hypertension, fatty liver, and cardiopulmonary disorders. Furthermore, childhood obesity is strongly associated with an increased risk of developing metabolic diseases, including type 2 diabetes, high cholesterol, and insulin resistance, all of which are precursors to more serious conditions later in life. Children with obesity are more likely to remain obese into adulthood, leading to a greater burden on adult health and an increased risk of premature death. In addition, due to their young age, they are easily discriminated against by peers of the same age, which can lead to psychological problems and affect their healthy growth (15–17). Therefore, doing a good job in the prevention and control of obesity is of great significance for preschool children. As childhood obesity rates continue to rise, healthcare systems will face increasing demands for medical services related to obesity-related diseases. The treatment of obesity and its associated comorbidities, such as heart disease, diabetes, and liver disease, places a substantial financial burden on healthcare systems worldwide. Additionally, the early onset of these health problems may lead to longer periods of healthcare utilization throughout an individual's life, contributing to higher healthcare costs and reduced quality of life.

This study found that out of the 910 children, there were 149 cases of obesity, accounting for 16.37% of all children, which is consistent with the results of Li Xiaozhen et al.'s study (18). When comparing the prevalence of obesity in this study to other regional or global data, we observe differences depending on geographical setting. For example, studies from urban areas in China report similar obesity rates, while rural areas tend to show lower prevalence rates (19). This difference may be attributed to variations in socioeconomic status, access to healthcare, and lifestyle factors such as diet and physical activity levels. Globally, childhood obesity rates in high-income countries such as the United States and the United Kingdom are generally higher, often exceeding 20%, while countries with lower income levels show lower rates of childhood obesity (20). These variations highlight the influence of cultural, environmental, and policy factors on childhood obesity prevalence.

Further analysis of the related influencing factors of obesity showed that high birth weight, high parental BMI, picky eating, preference for meat, high frequency of junk food intake, and binge eating were all risk factors for the development of obesity in preschool children, while high parental education level and longer daily outdoor exercise time were protective factors, in accordance with previous study (21). Based on the above results, the following intervention measures are proposed: for children with high birth weight and high parental BMI, more attention should be paid to obesity prevention and control; for parents, health education should be strengthened, and the importance of obesity and the need for a healthy diet should be actively promoted (22). Parents should provide children with a scientifically and rationally balanced diet (23), increase the proportion of vegetables, reduce the intake of fats and sugars, correct children's unhealthy eating habits (24), and stay away from junk food; children should be encouraged to participate in outdoor activities (25), increase physical exercise, and if conditions do not allow, indoor exercises should be conducted appropriately to maintain their body shape and prevent obesity.

Global policy recommendations, including those from the World Health Organization (WHO), highlight the importance of comprehensive, multi-level interventions to address childhood obesity. WHO emphasizes that early childhood is a critical period for physical and cognitive development, during which children's habits, including those related to physical activity, screen time, and sleep, are formed. WHO recommends policies to reduce the marketing of unhealthy foods to children, promote physical activity through urban planning that encourages active transportation, and improve school environments by providing healthier food options and enhancing physical education programs. Furthermore, WHO advocates for guidelines on physical activity, sedentary behavior, and sleep for children, emphasizing that daily physical activity should be balanced with adequate sleep and limited screen time. Governments are encouraged to collaborate with the private sector and non-governmental organizations to support public health campaigns, improve nutritional standards in schools, and ensure that children's physical activity guidelines are implemented effectively. Special attention should be given to the quality of sedentary time, with recommendations for non-screen-based, interactive activities like reading and storytelling to support cognitive and social development (26, 27).

Our findings are consistent with the existing literature, which indicates that high birth weight, high parental BMI, picky eating, a preference for meat, a high frequency of junk food intake and binge eating are risk factors for the development of obesity in preschool children (28–30). Additionally, we found that high parental education and longer daily outdoor activity time for children were identified as protective factors against obesity. These findings highlight the importance of lifestyle and environmental factors in obesity development. In Europe, WHO member states have implemented various measures to address childhood obesity. Prevention planning in Europe has focused on multisectoral cooperation, health-promoting schools, community involvement, and policy support (31). For instance, some countries have enacted laws restricting the advertising of unhealthy foods while others have promoted healthier food choices and physical activity in schools. Our findings underscore strategies similar to those used in European prevention planning by emphasizing parental education and increased physical activity among children. Effective obesity prevention measures require a combination of individual, family-based educational interventions along with policy-level initiatives. At an individual level, raising awareness about healthy eating habits and active lifestyles is crucial. Family-level interventions should include parental education and changes in lifestyle habits within the family unit. Educational interventions should focus on improving school environments by providing healthy meal options and incorporating physical education programs. Policy-level interventions should involve developing guidelines that promote healthy food environments and encourage physical activity.

This study has some limitations: (1) The present study selected preschool children who underwent physical examination in a specific hospital within a specific time range, which may limit the generalizability of the sample. (2) This study used a retrospective cohort study design, which cannot completely exclude potential confounding factors and information bias, but we tried to collect various information from the two groups of children and parents. (3) The study was conducted in a specific medical institution which may limit the generalizability of the findings and selection bias may also be a concern. (4) Since parents were required to reflect and recall information in the questionnaire survey, there is a potential for information bias due to parental recall. Memory biases, such as the tendency to overestimate or underestimate certain behaviors (e.g., junk food consumption or physical activity), can occur when parents try to recall specific details. This could lead to inaccuracies, especially for behaviors that vary over time or are harder to measure. Such biases may affect the accuracy of data on key variables, such as dietary habits, physical activity, and behaviors like picky eating or binge eating. Parents might have difficulty recalling the exact frequency of certain behaviors or may provide socially desirable answers, which could impact the findings related to parental factors (e.g., BMI) and childhood obesity. Future research can make up for these limitations by using more refined designs and larger samples in multicenter studies.

In conclusion, there are multiple risk factors for the development of obesity in preschool children, mainly including high birth weight, high parental BMI, picky eating, preference for meat, high frequency of junk food intake, and binge eating. Effective interventions need to be carried out for the above risk factors to reduce the occurrence of obesity and promote the healthy growth of children.

## Data Availability

The raw data supporting the conclusions of this article will be made available by the authors, without undue reservation.
